# Noisy galvanic vestibular stimulation improves vestibular perception in bilateral vestibulopathy

**DOI:** 10.1007/s00415-022-11438-8

**Published:** 2022-11-02

**Authors:** Max Wuehr, Josefine Eder, Aram Keywan, Klaus Jahn

**Affiliations:** 1 German Center for Vertigo and Balance Disorders (DSGZ), Ludwig-Maximilians-University of Munich, Marchioninistrasse 15, 81377 Munich, Germany; 2 Schön Klinik Bad Aibling, Bad Aibling, Germany

**Keywords:** Bilateral vestibulopathy, Galvanic vestibular stimulation, Stochastic resonance, Vestibular perception, Balance

## Abstract

**Background:**

Patients with bilateral vestibulopathy (BVP) suffer from impaired vestibular motion perception that is linked to deficits in spatial memory and navigation.

**Objective:**

To examine the potential therapeutic effect of imperceptible noisy galvanic vestibular stimulation (nGVS) on impaired vestibular perceptual performance in BVP.

**Methods:**

In 11 patients with BVP (mean age: 54.0 ± 8.3 years, 7 females), we initially determined the nGVS intensity that optimally stabilizes balance during a static posturographic assessment. Subsequently, effects of optimal nGVS vs. sham stimulation on vestibular motion perception were examined in randomized order. Vestibular perceptual performance was determined as direction recognition thresholds for head-centered roll tilt motion on a 6DOF motion platform in the absence of any visual or auditory motion cues.

**Results:**

For each patient, an nGVS intensity that optimally stabilized static balance compared to sham stimulation could be identified (mean 0.36 ± 0.16 mA). nGVS at optimal intensity resulted in lowered vestibular perceptual thresholds (0.94 ± 0.30 deg/s) compared to sham stimulation (1.67 ± 1.11 deg/s; *p* = 0.040). nGVS-induced improvements in vestibular perception were observed in 8 of 11 patients (73%) and were greater in patients with poorer perceptual performance during sham stimulation (*R* = − 0.791; *p* = 0.007).

**Conclusions:**

nGVS is effective in improving impaired vestibular motion perception in patients with BVP, in particular in those patients with poor baseline perceptual performance. Imperceptible vestibular noise stimulation might thus offer a non-invasive approach to target BVP-related impairments in spatial memory, orientation, and navigation.

## Introduction

Bilateral vestibulopathy (BVP) is characterized by a chronic reduced or absent bilateral vestibular function [1]. Patients primarily suffer from postural imbalance during standing and walking that worsens in darkness or on uneven ground and blurred vision induced by head movements (i.e., oscillopsia) [2–4]. Beyond, their impaired perceptual registration of head orientation and motion in space has been linked to deficits in spatial memory and navigation [5–7]. BVP-related symptoms considerably impact patients' daily activities and mobility [8], are associated to reduced quality of life [9] and an increased risk of recurrent falling [8, 10]. The general long-term prognosis of BVP is poor [11], and available treatment options are currently limited to physical therapy that can yield, if any, only partial compensation for lost vestibular function [12].

A majority of patients with BVP typically retain residual vestibular excitability and function [13, 14]. Within recent years, attempts have been made to augment and boost residual vestibular excitability in BVP by means of an imperceptible vestibular noise stimulation using non-invasive noisy galvanic vestibular stimulation (nGVS) [15, 16]. The rationale behind these attempts is stochastic resonance (SR)—a phenomenon according to which (pathologically increased) thresholds for sensory information processing can be lowered by application of an appropriate amount of low-intensity sensory noise [17, 18]. Previous studies could demonstrate that treatment with nGVS effectively improves vestibulospinal function [19, 20] and as a result stabilizes impaired balance [21, 22] and gait performance [23, 24] of patients with BVP. While nGVS-related treatment attempts in BVP so far focused on impaired postural regulation, recent evidence from healthy individuals indicates that nGVS might also directly affect vestibular perceptual performance [25–28] and could thus be effective to treat BVP-related deficits in spatial cognition.

In the current study, we examined the effects of imperceptible nGVS vs. sham stimulation on vestibular perceptual performance in patients with BVP. We demonstrate that nGVS effectively improves vestibular motion perception in particular in those patients with poor baseline perceptual performance. nGVS might thus provide a non-invasive and well-tolerated treatment option to target a wide range of BVP-related symptoms including non-motor deficits in spatial memory and navigation.

## Methods and materials

### Standard protocol approvals, registrations, and patient consents

The study protocol was approved by the ethics committee of the University of Munich (study ID: 20-1137) and registered at DRKS (DRKS00024660). Each patient gave written informed consent prior to participation.

### Participants

Eleven patients with BVP (mean age: 54.0 ± 8.3 years, 7 females) participated in the study. All patients showed a clinically proven deficit, i.e., a bilateral pathological video head impulse test (vHIT, horizontal gain < 0.6) and/or bilateral reduced or absent caloric responses (sum of maximal peak velocities of the slow-phase nystagmus with cold and warm water < 6 deg/s) [1]. Detailed clinical characteristics of patients are presented in Table 1.

**Table 1:** Clinical characteristics, stimulation characteristics and effects

Patient	Sex	Age	Etiology	Caloric response, deg/s^a^		vHIT gain		nGVS, mA	Perceptual threshold, deg/s	
				Left	Right	Left	Right		Sham	nGVS
P1	m	46	Genetic	4.1	5.2	0.04	0.12	0.2	0.71	1.05
P2	f	57	Idiopathic	2.6	2.4	0.74	0.67	0.7	4.34	1.31
P3	f	52	Idiopathic	4.4	2.6	0.18	0.16	0.6	1.89	0.81
P4	m	54	Idiopathic	2.7	0.7	0.32	0.22	0.2	1.25	1.35
P5	m	54	Idiopathic	3.0	4.9	0.18	0.02	0.2	0.73	0.71
P6	f	55	Genetic	5.7	2.5	0.22	0.15	0.4	2.72	1.15
P7	f	63	Idiopathic	2.5	4.4	0.11	0.03	0.4	1.62	1.20
P8	f	60	Ototoxic	13.8	7.4	0.31	0.32	0.3	2.08	0.69
P9	f	65	Idiopathic	3.3	2.6	0.66	0.34	0.4	0.39	0.56
P10	m	37	Idiopathic	7.5	12.0	0.53	0.15	0.2	1.11	1.03
P11	f	45	Ototoxic	3.5	3.3	0.30	0.26	0.3	1.49	0.52

*vHIT* video head impulse test, *nGVS* noisy galvanic vestibular stimulation

^a^Sum of maximal slow phase eye velocity during warm and cold caloric irrigation

### Galvanic vestibular stimulation

Vestibular noise stimulation (i.e., nGVS) was applied via a pair of 4.0 cm × 6.0 cm Ag–AgCl electrodes attached bilaterally over the left and right mastoid process. Zero-mean Gaussian white noise stimulation with a frequency range of 0–30 Hz and varying peak amplitudes of 0–0.7 mA was delivered by a mobile constant current stimulator (neuroConn®, Illmenau, Germany).

### Experimental procedures

The experimental procedures consisted of two parts: Initially, for each patient the nGVS intensity that optimally stabilized body balance as assessed by static posturography was individually identified. In the main part, the effect of nGVS at this optimal intensity on vestibular motion perception was assessed in comparison with sham stimulation (i.e., nGVS at 0 mA).

The initial identification of optimal nGVS intensity was performed in analogy to previous procedures [22, 28]: Body sway was recorded for 30 s on a posturographic force plate (Kistler, 9261A, Kistler Group, Winterthur, Switzerland) at 40 Hz while patients were standing with their eyes closed. This procedure was repeated eight times, while patients were stimulated with a different amplitude of nGVS (ranging from 0–0.7 mA, in a pseudo-randomized order) in each trial. Patients were blinded to the stimulation order and were given short breaks between trials to recover. Body sway during each trial was characterized by three different body sway measures: the mean velocity of the center of pressure (CoP) motion, the root mean square of CoP movement, and the envelopment area traced by the CoP [22]. The optimal nGVS intensity was determined as the one that yielded greatest reduction in all three body sway measures compared to sham stimulation (i.e., nGVS at 0 mA).

**Fig. 1:**
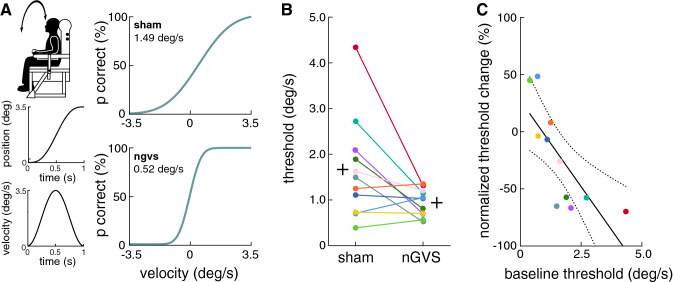
Effects of nGVS on vestibular perceptual thresholds for head-centered roll tilt motion. (**A**) Left panel: Vestibular perceptual thresholds were determined on a 6DOF motion platform with patients secured in a platform-mounted chair. Arrow indicates the rotational axis alongside the typical displacement and velocity profile of motion stimuli applied during psychophysical testing. Right panel: Exemplary psychometric curves of perceptual performance and corresponding thresholds during sham (i.e., nGVS at 0 mA) and optimal nGVS (patient P11). (**B**) Group effects of nGVS on perceptual thresholds revealed an improved perceptual performance compared to sham stimulation (*p* = 0.04; black crosses represent the group average for each condition). (**C**) Higher baseline perceptual thresholds during sham stimulation were associated to greater nGVS-induced improvements in perceptual performance (*R* = − 0.791; *p* = 0.007)

In the main part, effects of nGVS at optimal intensity on vestibular perceptual thresholds were examined. Vestibular perceptual thresholds were determined as direction recognition thresholds (DRT) for head-centered roll tilt motion in analogy to previous procedures [25–28]. Perception of this motion requires the integration of cues from the semicircular canals and the otoliths of the peripheral vestibular endorgans [29]. Patients were secured in a chair mounted on 6DOF motion platform (Moog 6DOF2000E, East Aurora, New York) by a five-point harness and an adjustable head restraint. The experiment was performed in total darkness and patients wore noise-cancelling headphones to minimize the presence of non-vestibular sensory cues [30]. The complete procedure comprised 150 trials. Each trial consisted of a head-centered roll tilt motion made of a single half-cycle acceleration that followed a raised-cosine velocity profile (Fig. 1A) at 1 Hz to either the left or right (in randomized order), and patients had to indicate the direction of perceived motion by button press. The peak motion velocity of each trial was varied following an adaptive 3-down 1-up staircase procedure. Subsequently, a cumulative Gaussian psychometric curve was fitted to the response data of all trials and the resultant DRT was determined as the magnitude of roll tilt velocity, which could be distinguished at a rate of 79.4% [31]. DRTs were determined in two sessions, once during nGVS delivered at optimal intensity and once during sham stimulation (i.e., nGVS at 0 mA) in a pseudo-randomized order. Patients were blinded to the stimulation order and were given an extended break to recover in-between sessions.

### Data and statistical analysis

Data are reported as mean ± SD. Effects of nGVS on vestibular perceptual thresholds were examined using a one-way repeated measures analysis of variance (ANOVA) with the factor stimulation (nGVS vs. sham). Pearson's correlations were performed to test for any association between clinical test outcomes (caloric response, vHIT gain), baseline thresholds, and nGVS-induced changes in thresholds. Results were considered significant at *p* < 0.05. Statistical analysis was performed using SPSS (Version 26.0, IBM Corp., USA).

### Data availability

Data reported in the article will be shared by any appropriately qualified investigator on request after pseudonymization.

## Results

Administration of nGVS at intensities ranging from 0–0.7 mA was well tolerated and did not cause disequilibrium in any of the examined patients. For each patient, we identified an optimal nGVS intensity at which static balance was effectively stabilized (optimal nGVS intensity mean 0.36 ± 0.16 mA, range 0.2–0.7, Table 1). Compared to sham stimulation, stimulation at optimal nGVS reduced body sway velocity by 25 ± 14%, the root mean square of body sway by 22 ± 18%, and body sway area by 32 ± 26%. Stimulation at optimal nGVS intensity was not perceived by any patient.

Psychophysical assessment of baseline perceptual thresholds for head-centered roll tilt motion during sham stimulation (i.e., nGVS at 0 mA) yielded an average threshold level of 1.67 ± 1.11 deg/s, which closely corresponds to the range of previously reported thresholds in patients with BVP [32]. Baseline perceptual thresholds of patients were not associated to any of the clinical outcomes from vestibular function tests (i.e., caloric response or vHIT gain).

Application of nGVS at optimal intensity resulted in lowered perceptual thresholds (*F*_1,10_ = 5.58; *p* = 0.040; effect size: *η*^2^_p_ = 0.36) in 8 of 11 patients (73%, Fig. 1B). Vestibular thresholds during nGVS were found at 0.94 ± 0.30 deg/s corresponding to an average improvement of 23 ± 44%. Treatment effects of nGVS were not associated to the degree of vestibular hypofunction as assessed by clinical vestibular function tests. However, the degree of nGVS-induced threshold reductions was correlated with higher baseline perceptual thresholds determined during sham stimulation (*R* = − 0.791; *p* = 0.007; Fig. 1C).

## Discussion

In this study, we examined the potential therapeutic effects of imperceptible, low-intensity vestibular noise stimulation (i.e., nGVS) on impaired vestibular perceptual capacity in patients with BVP. Vestibular perceptual performance was assessed by means of an established psychophysical two-alternative forced choice paradigm that has been shown excellent test-to-retest reliability [31, 33]. In more than two-third of patients, we observed that application of nGVS effectively lowered vestibular thresholds for the perception of head-centered roll tilt stimuli. In particular those patients with poor vestibular perceptual performance at baseline (i.e., during sham stimulation) notably benefited from nGVS treatment.

The presumed mechanism underlying the observed therapeutic effect is SR [20, 25]. According to this phenomenon, addition of an appropriate amount of noise to a sensory system can effectively lower the system's threshold for signal processing whereas to low or high noise will either not affect or disturb signal transfer [17, 18]. By applying a wide range of nGVS amplitudes (0–0.7 mA) in young healthy individuals, Galvan-Garza and colleagues could previously demonstrate SR-like modulations of vestibular perception with optimal enhancements at intermediate nGVS intensities (0.3–0.5 mA) in 78% of the examined individuals [25]. The present observations in patients with BVP closely correspond to this previous report, both in terms of the rate of responders (73% vs. 78%) and the overall magnitude of response (23% vs. 25% improvement). Such SR-like enhancements of perceptual capacity are not limited to the vestibular system, but have been previously analogously demonstrated for human visual [34], auditory [35], and tactile perception [36].

BVP has been consistently associated with pathologically increased vestibular perceptual thresholds for the registration of translational and rotational motion stimuli [32, 37–39]. Compared to previously reported perceptual thresholds for roll tilt motion in healthy individuals in their sixth decade of life (mean: 1.19 deg/s; 95% CI 1.00–1.42 deg/s [40]), baseline perceptual thresholds in our cohort of patients (mean: 1.67 deg/s; 95% CI 0.92–2.42 deg/s) were in average increased by 40%. Impaired vestibular perceptual performance has been suggested to contribute to a variety of motor and non-motor symptoms associated with BVP [41]. Accordingly, vestibular contributions to balance control are not confined to vestibulospinal reflex control of upright posture but also involve perceptual registration of head and body orientation in space [42, 43]. In the elderly, increased roll tilt perceptual thresholds have been associated to deficits of balance control while standing with eyes closed on compliant support surface [40, 44]—a condition that specifically challenges vestibular balance regulation. The present and previous research in patients with BVP suggests that treatment with nGVS simultaneously targets the vestibular perceptual and the vestibulospinal reflex level [19] and both effects presumably contribute to the reported stabilizing effect of nGVS on static and dynamic balance in BVP [21–24].

Beyond imbalance, there is a range of non-motor symptoms associated with BVP that might specifically benefit from nGVS-induced improvements of vestibular perceptual capacity. The impaired monitoring of head-in-space orientation and motion in BVP is typically accompanied by specific impairments of navigation, spatial learning and memory as examined in virtual [5, 45] and real space [7] navigation tests. These deficits extend to limitations in patient’s activities of daily living in terms of frequent experiences of spatial disorientation, misjudgments of distances, and an increased spatial anxiety [6, 45]. It is conceivable that a sensitization of vestibular perception by nGVS might specifically ameliorate deficits of spatial memory, orientation, and navigation in BVP. In line with this assumption, a previous study could demonstrate nGVS-induced enhancements of spatial memory during a virtual navigation task in young healthy adults [46]. Further studies using virtual and/or real space test paradigms of navigation are, however, required to explore to potential impact of nGVS treatment on deficits of spatial memory and navigation in patients with BVP.

Certain limitations of this study have to be considered. Due to the lengthy procedure of the psychophysical examination and in accordance to previous studies [26–28], we did not examine stimulation effects on vestibular perceptual performance across a range of varying nGVS levels but only tested one nGVS intensity that was individually determined beforehand using a posturographic task. It is conceivable that nGVS intensities that optimally stabilize static posture may more or less differ from those that have the greatest benefit on vestibular motion perception [28]. Hence, an evaluation of different nGVS levels on perceptual performance may have resulted in an even higher rate of responders and magnitude of response as currently observed. Secondly, we only evaluated stimulation effects on one axis of motion, i.e., head-centered rotation in the roll plane. In accordance to previous studies, we specifically focused on perceptual performance along this axis since it involves the integration of sensory cues from both vestibular endorgan structures (semicircular canals and otoliths) [29] and is closely linked to balance performance [40, 44]. Due to the non-specific nature of the nGVS stimulus, we would expect analogous benefits of nGVS on perceptual performance along other axis of motion. However, future studies in patients with BVP are required to verify this assumption—in particular for perceptual performance in the horizontal plane that is essential for spatial orientation and navigation [47]. Finally, while our psychophysical paradigm focused on the perception of vertical canal and otolith cues, clinical evaluation of vestibular hypofunction was primarily based on horizontal canal function. This discrepancy might hence explain the lack of observed association of nGVS treatment effects on roll tilt perceptual thresholds and clinical measures of vestibular hypofunction.

In conclusion, we provide evidence that non-invasive and imperceptible vestibular noise stimulation is effective in improving impaired vestibular perceptual performance in patients with BVP, in particular in those patients with poor baseline perceptual performance. Future studies are required to explore further behavioral consequences of this therapeutic effect, in particular with respect to BVP-related deficits of spatial memory and navigation.
